# Treatment outcome of localized prostate cancer using transperineal ultrasound image-guided radiotherapy

**DOI:** 10.1186/s13014-024-02490-x

**Published:** 2024-08-01

**Authors:** Kenji Takai, Ryota Watanabe, Ken-ichi Hyogo, Yuri Ito, Nobuko Minagawa, Yusuke Sato, Yoshikazu Matsuda, Kenji Nemoto

**Affiliations:** 1 Department of Radiology, Yamagata City Hospital Saiseikan, Yamagata, Japan; 2 Central Radiology Center, Yamagata City Hospital Saiseikan, Yamagata, Japan; 3 Department of Radiation Oncology, Yamagata University, Yamagata, Japan

**Keywords:** Prostate cancer, Transperineal, Ultrasound, Real-time monitoring, Image-guided radiotherapy, TPUS, IGRT, PSA, Toxicity

## Abstract

**Background:**

We report the results of a retrospective analysis of localized prostate cancer (LPCa) treated with transperineal ultrasound image-guided radiotherapy (TPUS-IGRT).

**Methods:**

A total of 124 patients (median age: 74 y, 46–84 y) with LPCa who underwent TPUS-IGRT (Clarity Autoscan system; CAS, Elekta; Stockholm, Sweden) between April 2016 and October 2021 for curative/after hormone induction were enrolled. The number of patients by risk (National Comprehensive Cancer Network 2019) was 7, 25, 42, and 50 for low (LR), good intermediate (good IR), poor intermediate (poor IR), and high (HR)/very high (VHR), respectively. Ninety-five patients were given neoadjuvant hormonal therapy. The planning target volume margin setting was 3 mm for rectal in most cases, 5–7 mm for superior/inferior, and 5 mm for anterior/right/left. The principle prescribed dose is 74 Gy (LR), 76 Gy (good IR), and 76–78 Gy (poor IR or above). CAS was equipped with a real-time prostate intrafraction monitoring (RTPIFM) system. When a displacement of 2–3 mm or more was detected, irradiation was paused, and the patients were placed on standby for prostate reinstatement/recorrection. Of the 3135 fractions in 85 patients for whom RTPIFM was performed, 1008 fractions (32.1%) were recorrected at least once after starting irradiation.

**Results:**

A total of 123 patients completed the radiotherapy course. The 5-year overall survival rate was 95.9%. The 5-year biological prostate-specific antigen relapse-free survival rate (bPFS) was 100% for LR, 92.9% for intermediate IR, and 93.2% for HR/VHR (Phoenix method). The 5-year late toxicity rate of Grade 2+ was 7.4% for genitourinary (GU) and 6.5% for gastrointestinal (GI) organs. Comparing the ≤ 76 Gy group to the 78 Gy group for both GU and GI organs, the incidence was higher in the 78 Gy group for both groups.

**Conclusion:**

These results suggest that TPUS-IGRT is well tolerated, as the bPFS and incidence of late toxicity are almost comparable to those reported by other sources of image-guided radiotherapy.

**Supplementary Information:**

The online version contains supplementary material available at 10.1186/s13014-024-02490-x.

## Background

External beam radiotherapy (EBRT) is widely used as one of the curative therapies for localized prostate cancer (LPCa), and high doses are administered to optimize tumor control. The results of biological prostate-specific antigen relapse-free survival (bPFS) have been reported to be comparable to those of total prostatectomy [1, 2]. On the other hand, intrafractional prostate motion (IntraPM) and interfractional prostate motion (InterPM) during irradiation have been problems in EBRT, and image-guided radiotherapy (IGRT) has been used as a countermeasure. There are reports that IGRT can reduce toxicity caused by EBRT [3, 4], and IGRT has been introduced as a technique that allows a safe dose increase of approximately 10% [5]. Zelefsky et al. performed a study using IGRT with 86.4 Gy and reported significantly reduced late urologic toxicity and significantly better bPFS in the high-risk (HR) group [6]. IGRT modalities include the insertion of gold markers [7–9] or electromagnetic transponders (EMT) [10] into the prostate to monitor and correct the position of the markers and the use of a linear accelerator (Linac) that possesses a magnetic resonance imaging (MRI) guiding system [11]. Other modalities include the Clarity Autoscan system (CAS) (Elekta, Stockholm, Sweden, Supp A1) as an IGRT system that can monitor prostate position in real time during EBRT, is noninvasive and low-cost, and can be implemented into conventional EBRT systems. The positional accuracy of CAS has been reported to be comparable to that of other monitoring systems [12]. Although reports of stereotactic body radiotherapy (SBRT) [13, 14] have been published, there are currently very few reports on the outcomes of conventional dose-fractionated EBRT (CDFRT).

We have implemented CAS at Yamagata City Hospital Saiseikan (YCHS) and report the results of a retrospective analysis of the outcomes: 5-year overall survival (OS), bPFS, and toxicity of CDFRT with ultrasound image-guided radiotherapy (TPUS-IGRT) for LPCa. CDFRT has many more long-term results than SBRT, and we believe it is worthwhile to evaluate the use of TPUS-IGRT in CDFRT.

## Method

### Our study was reviewed and approved by the Ethics Committee of YCHS

#### Patients

In our study, which is a retrospective analysis, 124 patients with LPCa diagnosed as cT1-4N0M0 who started radical radiotherapy with CAS between April 2016 and October 2021 in YCHS were enrolled. All 124 patients were pathologically confirmed by prostate biopsy, and lymph node/distant metastases were excluded by computed tomography (CT)/MRI. On the other hand, N1/M1 patients, patients for salvage of hormonal therapy (HTx)/chemotherapy resistance and patients for relapse prevention after total prostatectomy were excluded.

The median age was 74 (46–84) years, the median prostate-specific antigen (PSA) level (ng/ml) at first diagnosis was 7.622 (2.843–300), 76 (61.3%) were < 10, 32 (25.8%) were 10–20, and 16 (12.9%) were > 20.

The breakdown of the 124 patients in the analysis by risk classification according to the National Comprehensive Cancer Network (NCCN) 2019 was: low risk (LR) 7 patients, intermediate risk (IR) 67 patients (good prognosis; favorable IR: 25 patients, poor prognosis; poor IR: 42 patients), HR 39 patients, and very high risk (VHR) 11 patients.

The medical history included 17 patients on anticoagulants, 19 patients with diabetes, 2 patients on hemodialysis (HD), and 1 patient with rheumatoid arthritis (RA). Patient details are shown in Table 1a.

**Table 1:** Patients and TPUS-IGRT setup details

(a) Patients characteristics		n	%
Total patients	Median 74y(46-84y)	124	–
T stage	T1/T2/T3/T4	11/101/11/1	8.9/81.5/8.9/0.8
GSG	1/2/3/4/5	23/30/24/29/18	18.5/24.2/19.4/23.4/12.5
initial PSA (ng/ml)	< 10/10–20/ > 20	76/32/16	61.3/25.8/12.9
NCCN 2019 risk Group	LR/good IR/poor IR/HR/VHR	7/25/42/39/11	5.6/20.3/33.9/31.5/8.9
Past history	Anticoagulants	17	13.7
	Diabetes	19	15.3
	HD	2	1.6
	RA	1	0.8

(b) *PTV margin setup, fractions with/without RTPIFM*						
RTPIFM		None		Part of EBRT course^a^	Almost all of EBRT course^b^	Total n
n of patients		39		6	79	124
*PTV margin setup*	SI\rectal (mm)	5	3	3	3	Total
	5	–	–	–	68	68
	7	7^c^	32	6	11	56
	Total n	7^c^	–	117		124
Number of fractions without RTPIFM (N/total, %)		1591 (33.7)				Total fractions
Number of fractions: Recorrected at least once among fraction with RTPIFM/Total with RTPIFM(%)^d^				1008/3135 (32.1)		4726

^a^Displacement criterion for recorrection ≧ 2–3 mm. 104 fractions out of 228 fractions (45.6%)

^b^Displacement criterion for recorrection ≧ 2 mm. 3031 fractions out of 3042 fractions (99.6%)

^c^Two of them changed to the 3 mm rectal side during the EBRT course

^d^The RTPIFM was operational after Nov. 21, 2017, following approval for legal use

TPUS-IGRT, transperitoneal ultrasound image-guided radiotherapy; GSG, Gleason score group; PSA, prostate-specific antigen; NCCN, National Comprehensive Cancer Network; LR, low risk; IR, intermediate risk; HR, high risk; VHR, very high risk; HD, hemodialysis; RA, rheumatoid arthritis; PTV, planning target volume; RTPIFM, real-time prostate intrafractional monitoring; SI, superior-inferior; EBRT, external beam radiotherapy

#### Radiotherapy planning/patient setup for Linac

To maintain a similar bladder condition, patients are required to urinate approximately 30 min to 1 h prior to CT for TPUS-IGRT planning and each irradiation and then to drink 200–500 ml of water and store it until the end of the examination/treatment. No enema is used to promote defecation.

Treatment planning images are taken, and irradiation is performed with the patient in the supine position, secured in the CAS-attached fixation device, and the probe attached to the perineum. For treatment planning, the CAS system was brought into the CT (GE, BrightSpeed®) room, and CT imaging was followed by transperineal ultrasound imaging. The origin on the image coordinates was marked on the abdominal skin surface. A slice thickness of 2.5 mm was used for CT imaging. The treatment planning workstation (WS) was Pinnacle (Philipps, Netherlands), and the TPUS images were transferred to the WS and fused with the CT images to create the contour of each organ. The prostate was defined as the reference positioning volume (RPV).

The clinical target volume (CTV) was defined as follows: the CTV of the tumor area (CTV1) was the prostate, plus the capsular invasion area for cT3a, the seminal vesicle invasion area for cT3b, and the tumor invasion area for cT4. In addition, the root of the seminal vesicle (approximately 1/3 of the bilateral seminal vesicles on the prostatic side) is defined as the prophylactic CTV (CTV2) for patients at risk of good IR or higher. The isocenter (IC) was defined as approximately the center of gravity of the RPV.

The planning target volume (PTV) was set by 3D expansion of CTV1/2. At the beginning of the introduction of CAS, it was set wider, but after some time after the introduction, it was set narrower.

The PTV margin settings were 5 mm (68 patients)/7 mm (56 patients) for the superior-inferior side (SI), 3 mm (117 patients)/5 mm (7 patients, 2 of whom were initially 5 mm → changed to 3 mm during the process) for the rectal side, and 5 mm for the right-left side (RL) and anterior side in all patients (Table 1b).

Linac was Synergy (Elekta, Stockholm, Sweden), and 10 MV X-rays and 4–5 coplanar beams were used in principle.

The patient was set up on the Linac bed with the probe attached to the perineum in the same position as during CT imaging, and the origin on the skin marker was used as a landmark. The IC was aligned with the center of the gantry rotation axis of the Linac. Next, TPUS was performed before irradiation, the position of the RPV was corrected, and irradiation was started using that position as the baseline.

#### Use of real-time prostate intrafractional monitoring (RTPIFM)

YCHS has been operating CAS since April 2016. However, due to the circumstances of the approval of the use of the device by Japanese law, we only corrected the position of the fraction just before irradiation and did not perform real-time prostate intrafractional monitoring (RTPIFM) when the CAS was first introduced. RTPIFM was not performed in 39 patients, RTPIFM was introduced in 6 patients in the middle of the TPUS-IGRT period, and the entire treatment period was included in the RTPIFM operation period for the other 79 patients (Table 1b).

RTPIFM monitors the RPV over time during beam irradiation, acquiring TPUS images at a rate of 3–4 frames per second to monitor the RPV position in real time (Supp. A2 for RTPIFM screen description.). When the RPV is displaced more than 2–3 mm in either direction from the baseline, the beam is paused, the patient waits for spontaneous recovery, and irradiation is resumed when the RPV has recovered. If the displacement from the baseline continued for more than 5 s even after the beam irradiation of one of the ports was completed or if the displacement was expected to continue for more than 10 s during the irradiation of one of the ports, the position was recorrected, and the irradiation was continued. When RTPIFM was first introduced, the displacement criterion was set to "2–3 mm or more" (6 patients), but in the 79 patients where RTPIFM was performed almost every time, the criterion was set to “2 mm or more”.

Of the 85 patients and 3135 fractions for whom RTPIFM was performed, 1008 fractions (32.1%) were recorrected at least once after the start of irradiation (Table 1b).

#### Prescribed/administered dose

The principle prescribed doses were 74 Gy/37 fr for LR, 76 Gy/38 fr for good IR, and 76 Gy/38 fr (-June 2018) or 78 Gy/39 fr (July 2018-) for poor IR or above for CTV1 with IC prescription. In addition, 50 Gy/25 fr was prescribed for CTV2. No whole pelvis/small pelvis irradiation was performed in the cases analyzed in our study.

All but 3 patients were able to receive the prescribed dose in principle (97.6% achievement rate). Doses by risk group were 74 Gy/37 fr for all 7 patients in the LR group, 76 Gy/38 fr for 24 patients and 74 Gy/37 fr for 1 patient in the good IR group (LR was initially diagnosed but was changed after reevaluation of staging after treatment). In the poor IR group, the dose was 78 Gy/39 fr for 14 patients, 76 Gy/38 fr for 27 patients and 68.4 Gy/38 fr for 1 patient (dose reduced due to a history of RA). In the HR group, 23 patients had 78 Gy/39 fr and 15 patients 76 Gy/38 fr, and in the VHR group, 7 patients had 78 Gy/39 fr and 4 patients 76 Gy/38 fr. In addition, one patient (HR) developed subarachnoid hemorrhage (SAH) during the treatment period and discontinued treatment at 32 Gy/16 fr (Supp. B).

#### HTx

HTx was decided at the discretion of the attending urologists. Neoadjuvant HTx (NAHT) was performed in 95 patients, including LR: 1 (/7 = 14.3%), good IR: 15 (/25 = 60.0%), poor IR: 33 (/42 = 78.6%), HR: 35 (/39 = 89.7%), and VHR: 11 (/11 = 100%). Of these, 92 were performed in combination with TPUS-IGRT; in one case, HTx was started almost simultaneously with the start of TPUS-IGRT. HTx combined with EBRT was used in 93 patients. Eighty-four (67.7%) had a PSA of < 1.0, 39 (31.5%) had a PSA of ≥ 1.0, and 1 was unknown at the most recent start of TPUS-IGRT. Of the 39 patients with PSA ≥ 1.0, 29 had not undergone NAHT. Adjuvant HTx (AHT) was performed in 85 patients after completion of EBRT, ranging from 1 to 65 months (median 13 months). Twenty-seven patients received 1–6 months, and 58 patients received > 6 months (Supp. C).

#### Follow-up after TPUS-IGRT/data analysis

Progress after the TPUS-IGRT period was collected from medical records and other medical information. If the information could not be obtained from the medical record, it was collected by telephone contact with the patient or by paper questionnaire. The final month of follow-up was February 2023. Patients could be followed up except for one patient who developed SAH during TPUS-IGRT and discontinued treatment (HR group; treatment was discontinued at 32 Gy/16 fr).

PSA relapse was defined according to the Phoenix determination method [15].

Toxicity was determined according to the Common Terminology Criteria for Adverse Events (CTCAE) Version 4.0 (by the National Cancer Institute).

OS, bPFS and genitourinary (GU)/gastrointestinal (GI) organ late toxicity (≥ grade 2) incidence curves were generated using Kaplan‒Meier analysis.

Univariate (UA) and multivariate (MA) analyses were performed for factors of PSA relapse and late GU/GI toxicity (≥ Grade 2). The χ^2^ test or Cox regression model was used for UA, and the Cox regression model was used for MA. The factors for PSA relapse were age, T stage, Gleason score group (GSG), PSA level at initial diagnosis, PSA level at the start of EBRT, NAHT duration, PTV margin, RTPIFM status (6 patients who started RTPIFM in the middle of EBRT duration were included in the "no" category), and AHT duration. Factors for late toxicity included age, history of anticoagulant use, history of diabetes, NAHT duration, PTV margin, RTPIFM status, NAHT duration, presence or absence of acute onset of Grade 2 or higher, and AHT duration.

Significance difference tests were considered significant at *p* < 0.05.

## Result

We present the results of the analysis of 123 patients, excluding one patient who discontinued TPUS-IGRT due to SAH (no RTPIFM for all fractions).

### OS/bPFS

The median observation period was 47 months (3–80 months).

**Fig. 1:**
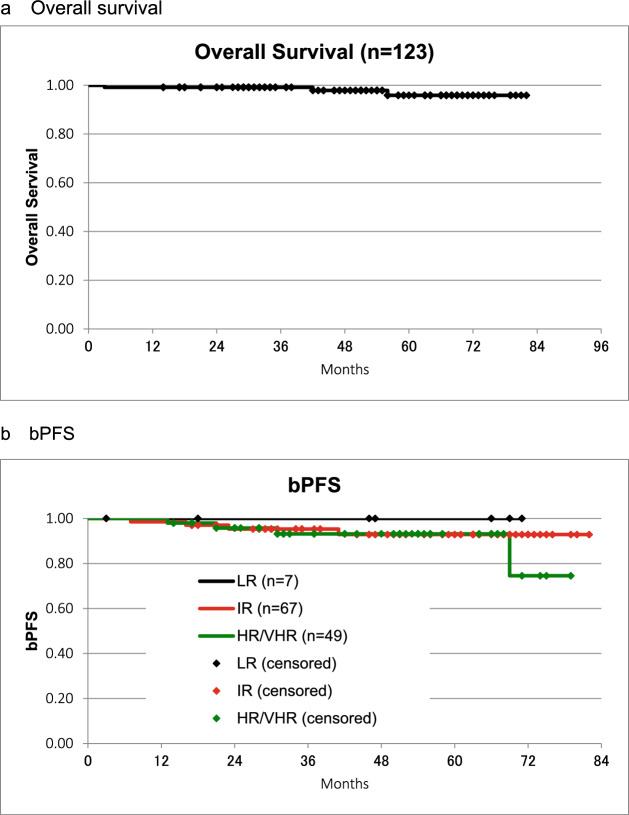
**a** An overall survival. 5y: 95.9%; No patients had cause-specific death. **b** bPFS. 5y: bPFS; LR: 100%; IR: 92.90%; HR/VHR: 93.20%; *p* = 0.6747 (log-rank test). bPFS, biological prostate-specific antigen relapse-free survival; LR, low risk; IR, intermediate risk; HR, high risk; VHR, very high risk

The 5-year OS was 95.9% (Fig. 1a), and no patient died of the current disease.

The 5-year bPFS by risk was LR: 100%, IR: 92.9%, and HR/VHR: 93.2%. (*p* = 0.6747, log-rank test) (Fig. 1b).

Factors of PSA relapse were analyzed by age (< 75 y vs ≥ 75 y), T stage, Gleason score group (GSG, ≤ 2 vs. ≥ 3), PSA at diagnosis, PSA immediately before TPUS-IGRT (< 0.1 vs ≥ 0.1), NAHT duration, SI margin (5 mm vs 7 mm), RTPIFM status, and AHT duration. Only "PSA at diagnosis" was significant for both UA/MA (UA: *p* = 0.0098, MA: *p* = 0.0106), while the other factors were not significant (Table 2).

**Table 2:** Statistical analyses of predictors for the 5-year bPFS, *p* values

Factor	*p* value	
	UA	MA
Age (< 75y vs ≥ 75y)	0.1942	0.2236
T stage	0.1384	0.1569
GSG (≤ 2 vs ≥ 3)	0.0706	0.0933
PSA at diagnosis	0.0098	0.0106
PSA just before EBRT (< 0.1 vs ≥ 0.1, ng/ml)	0.1435	0.1745
NAHT duration	0.6936	0.6884
RTPIFM status (yes vs no)	0.3853	0.9248
AHT duration	0.3326	0.3536

The *p* values for which significant differences were detected are underlined

bPFS, biological PSA-free survival; NAHT, neoadjuvant hormonal therapy; AHT, adjuvant hormonal therapy; UA, univariate analysis; MA, multivariate analysis

Others as in Table 1

### Toxicity

The incidence of Grade 2 or higher acute toxicity was 15 (12.2%) for GU organs and 25 (20.3%) for GI organs.

**Fig. 2:**
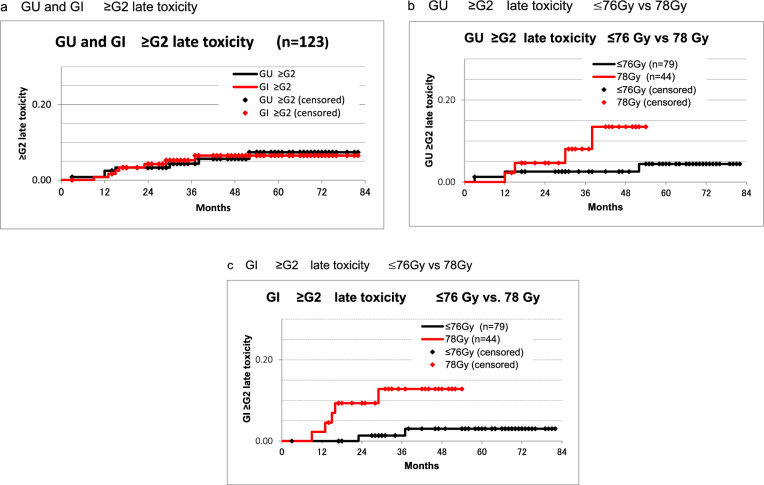
**a** GU and GI ≥ G2 late toxicity. Late toxicity rate 5y: GU 7.40%, GI: 6.50%. **b** GU ≥ G2 late toxicity ≤ 76 Gy vs 78 Gy. Late toxicity rate: ≤ 76 Gy 4.4% (5 y); 78 Gy 13.5% (4 y); *p* = 0.0922 Logrank test. **c** GI ≥ G2 late toxicity ≤ 76 Gy vs 78 Gy. Late toxicity rate: ≤ 76 Gy 3.0% (5 y); 78 Gy 12.8% (4 y); *p* = 0.0221 Logrank test. GU, genitourinary; GI, gastrointestinal; G2, grade 2

The 5-year incidence of Grade 2 or higher late toxicity was 7.4% in the GU organ and 6.5% in the GI organ (Fig. 2a). When analyzed by dose, the incidence rates were 4.4% at ≤ 76 Gy/5 years and 13.5% at 78 Gy/4 years for GU organs (*p* value: 0.0922 @ log-rank test, Fig. 2b), 3.0% at ≤ 76 Gy/5 years and 12.8% at 78 Gy/4 years for GI organs (*p* value: 0.0221 @ log-rank test, Fig. 2c). Both GU and GI were higher in the 78 Gy group, but significant differences were detected only in GI.

Three patients developed Grade 3, two of whom had rectal bleeding (9 and 15 months after completion of TPUS-IGRT) and one had erectile dysfunction (ED) 52 months after TPUS-IGRT. The two patients with rectal bleeding were both in the 78 Gy group and had been on HD, and both were treated with hyperbaric oxygenation (HBO) and recovered. One patient with ED was in the 76 Gy group and received hormone therapy for 53 months after TPUS-IGRT.

Factors for the development of late toxicity of Grade 2 or higher were age, presence of anticoagulants, presence of diabetes, NAHT duration, PTV margin (SI, 5 mm vs. 7 mm), RTPIFM status, early toxicity of Grade 2 or higher, and AHT duration. The results showed that late GU toxicity was significantly higher in cases of early toxicity of Grade 2 or higher (*p* value UA: 0.0037, MA: 0.0062) and in the AHT duration (*p* value UA: 0.0146, MA: 0.0121). In GI, the 5 mm margin group was analyzed as significantly higher in MA for the SI margin (vs. 7 mm) (*p* = 0.0303). No significant differences were detected otherwise (Table 3).

**Table 3:** Statistical analyses of predictors for late toxicity, *p* values

	GU, ≥ G2		GI, ≥ G2	
	*p* value		*p* value	
	UA	MA	UA	MA
Age	0.8583	0.8686	0.2372	0.1599
Anticoagulant	0.3145	0.3065	0.2753	0.2471
Diabetes	0.2831	0.2753	0.9302	0.8010
NAHT duration	0.8349	0.8247	NA	NA
SI margin (5 mm vs 7 mm)	0.8162	0.1691	0.0581	0.0303
RTPIFM status (Yes vs No)	0.8983	0.4800	0.222	0.1092
GU acute toxicity ≥ G2	0.0037	0.0062	NA	NA
AHT duration	0.0146	0.0121	NA	NA
GI acute toxicity ≥ G2	NA	NA	0.5767	0.7753

The *p* values for which significant differences were detected are underlined

GU, genitourinary; G2, grade 2; GI, gastrointestinal; NA; not applicable

Others as in Tables 1 and 2

## Discussion

### bPFS

In radical EBRT for prostate cancer, InterPM of the prostate is an issue in local control. Although we cannot compare the results of TPUS-IGRT in our study with those of patients in which TPUS-IGRT was not performed, at this time, we believe that the results of TPUS-IGRT in our study are generally comparable to those of other CDFRT with IGRT [7–9, 16] (Table 4). In our study, approximately 1/3 of the fractions that underwent RTPIFM underwent recorrection during RTPIFM. Despite the fact that the PTV margin was set as narrow as possible to reduce late rectal toxicity, the results are considered not inferior to other IGRT treatment outcomes. However, we consider it very difficult to evaluate whether the results of our study are superior to those reported by other researchers because of differences in patient background, HTx duration and concomitant use, and the proportion of each risk.

**Table 4:** Literature review: CDFRT using IGRT

Author	Risk	n	Dose (Gy)	HTx rate (%)	Modality of IGRT	IMRT rate (%)	Median F/U months	bPFS (PD, 5-year, %)	≥ G2 GU late toxicity (%)	≥ G2 GI late toxicity (%)
Takeda et al. [7]	IR	36	80 or 76	67	GM	100	60	100	6.4	6
	HR	105		95				82		
Martin JM et al. [8]	LR	59	79.8	13.6	GM	13 (total)	67.8	88.4	12.1	13.7
	IR	163		11				76.5		
	HR	37		45.9				77.9		
Kobayashi et al. [9]	IR	169	76	100	GM	100	43	95.9	8.9	5.5
	HR	133						87.2		
	VHR	23						73.1		
Tanaka et al. [16]	LR	205	74	59.2 (total)	MVCT	100	50	95.9	4.3	11.4
	IR	450	74 or 76					91.4		
	HR	345	78					85.5		
	VHR	91						80.2		
Current study	LR	7	74	14.3	TPUS	0	47	100	7.4(total) 4.4 (≤ 76 Gy) 13.5 (78 Gy)	6.5(total) 3.0(≤ 76 Gy)12.8(78 Gy)
	IR	67	68.4–78	73.1				92.9		
	HR/VHR	49 (38*/11)	76 or 78	91.8				93.2		

Abbreviations: CDHRT, conventional dose-fractionated radiotherapy; IGRT, image guided radiotherapy; HTx; hormonal therapy; GM, gold markers with on-board imager; MVCT, megavoltage computed tomography image-guided verification; TPUS, transperitoneal ultrasound; IMRT, intensity modulated radiotherapy; F/U. follow up; PD, Phoenix Consensus definition. Others as in Tables 1, 2, 3

*Number after excluding one patient who discontinued TPUS-IGRT

Several authors have reported a dose-dependent improvement in bPFS [17–23]. In our study, we compared the 4-year bPFS of patients treated with ≤ 76 Gy and 78 Gy in terms of poor IR and HR/VHR. Poor IR: 96.4% (n = 28) in the ≤ 76 Gy group and 85.7% (n = 14) in the 78 Gy group, *p* = 0.5169 (Supp D1). HR/VHR: 89.5% (n = 19) for the 76 Gy group and 95.0% (n = 30) for the 78 Gy group, *p* = 0.3769 (bPFS: Kaplan‒Meier method, *p* value: log-rank test) (Supp D2). In both groups, there was no significant difference. Given the relatively short median observation period of 47 months, more time will be needed to evaluate the dose dependence in the HR/VHR group. On the other hand, considering that the 5-year bPFS in the poor IR group was 96.4%, even in the 76 Gy group, 78 Gy may not be necessary in TPUS-IGRT.

### Late toxicity

Late toxicity of grade 2 or higher was in the 7% GU and 6% GI range, which is almost comparable to other IGRT reports [7–9, 16]. However, when compared to the group receiving ≤ 76 Gy and the group receiving 78 Gy, the latter was higher.

Regarding GI toxicity, the dosimetric factor of the rectum was not evaluated in our study. Rectal V70 (percentage of rectal volume irradiated with 70 Gy or more) has been shown to be a significant factor in the presence or absence of rectal injury [24, 25]. Our study did not employ intensity-modulated radiotherapy (IMRT), and it is possible that the V70 of the rectum in the 78 Gy group was higher than that assuming IMRT was employed. On the other hand, the incidence of grade 2 in the ≤ 76 Gy group was in the 3% range, which is comparable to or lower than other reports of IGRT [7–9, 16].

The MA of GI late toxicity showed that the SI margin was significantly higher in the 5 mm group than in the 7 mm group. This may be related to the fact that all 55 patients (one patient out of 56 was excluded from data analysis.) with a 7 mm SI margin received a dose of ≤ 76 Gy, while 44 of the 68 patients with a 5 mm margin received 78 Gy. In other words, the data from this study suggest that dose has a greater effect than SI margin on GI late toxicity for Grade 2 or higher.

Alicikus et al. [26] reported that the onset of GU acute grade 2 or higher was a statistical predictor of late GU grade 2 or higher, which is consistent with the results of our study.

Although there were no grade 4 or higher cases, two grade 3 patients had GI (HBO for rectal bleeding), and one had GU (ED). Both GI patients received 78 Gy with full RTPIFM and had a history of long-term HD due to chronic renal failure (CRF). In patients with CRF, there are reports [27, 28] suggesting pathologic changes in vascular abnormalities of colonic mucosal tissue. Considering the possibility of impaired tissue repair, we cannot find any reason to exclude the possibility of vulnerability to radiation tolerance in these two patients. In the present study, the use of RTPIFM in the case of the high dose of 78 Gy did not seem to avoid consequent damage, and a reduction in the prescribed dose may be necessary if IMRT is not used in long-term HD patients.

One case of ED was treated with 76 Gy without RTPIFM and continued AHT for 53 months after completion of TPUS-IGRT. EBRT [29] and HTx [30] have been cited as causes of ED as complications of LPCa treatment. One case of ED is thought to be possibly due to long-term HTx, although the influence of TPUS-IGRT cannot be ruled out.

### Significance of RTPIFM in InterPM and IntraPM

The prostate is known as an organ that often shows internal migration and displacement. To improve bPFS and reduce toxicity, it is considered useful to take measures to minimize displacement as much as possible and to use IGRT to correct the position of the displaced prostate.

Crevoisier et al. [31] reported a randomized study comparing the outcomes of CDFRT between a group that received IGRT once a week (n = 234) and a group that received IGRT every time (n = 236). IGRT was performed by cone-beam CT or ultrasound imaging immediately prior to irradiation for InterPM detection/correction. They reported significantly better bPFS and less late rectal toxicity in the latter group. Their report suggests that InterPM correction by repeated IGRT is useful for improving bPFS and reducing rectal toxicity.

To the best of our knowledge, there have been no comparative studies to determine whether IntraPM measures significantly differ in outcome or toxicity between CDFRT with and without IntraPM measures. In the case of SBRT, IntraPM countermeasures are also considered important because a large dose is administered per fraction. Here, it will be necessary to consider the significance of IntraPM measures in CDFRT, which has much larger fractions and smaller fraction size than SBRT.

Of the 4726 fractions in our study, 1591 fractions (33.7%) were without RTPIFM, and the actual status of IntraPM in those fractions is unknown. Of the 85 patients and 3135 fractions with RTPIFM in our study, 1008 fractions (32.1%) were recorrected at least once. Using this ratio, 511 fractions (32.1% of 1591 fractions without RTPIFM) had IntraPM to the extent that they needed to be recorrected. Those fractions would cause a "partial shot omission" inside the CTV, which could cause some areas of the prostate to be underdosed. It can cause a decrease in bPFS.

Tong et al. [32], in a study using EMT, found that the percentage of fractions with displacements of 5 mm or more for 30 s or longer was 4.6%, while the percentage of fractions with displacements of 3 mm or more for 30 s or longer was 27.2%. In a CAS study, Baker et al. [33] reported that the percentage of fractions with displacements of 2 mm or more in the RL/anterior–posterior/SI direction was 2%, 10%, and 4%, respectively, among 10 patients and 51 fractions. Richardson et al. [34] found that of 20 patients with 526 fractions treated with RTPIFM, an average of 24% of the total time was spent with a displacement of 3 mm or more in the rectal direction.

In YCHS, we only instructed patients to urinate 30 min to 1 h before treatment and drink 200–500 ml of water afterward and to store urine. The time and amount of water to store and drink depended on the patient's condition, and we did not establish a strict protocol to maintain good patient compliance with treatment.

The proportion of fractions recorrected by IntraPM in our study is likely to be comparable or higher than in the above three studies, but this may be related to the heterogeneity of pretreatment from patient to patient. Nevertheless, the bPFS seems generally comparable to other CDFRT reports [7–9, 16].

While CAS may contribute as a meaningful modality to maintain good bPFS while maintaining good patient compliance with treatment by modifying its position relative to InterPM, no significant differences in bPFS or late toxicity were detected between case groups with and without RTPIFM, although this is not a randomized study. However, from the viewpoint of dose uniformity to the prostate and reduction of rectal irradiation volume, we cannot immediately conclude the significance of RTPIFM in CDFRT at this point based on these results. The number of patients and follow-up period may not be sufficient to verify the significance of RTPIFM in our study, and it may be necessary to consider the effect of the different PTV margin margins (wider in the without RTPIFM group) in the with RTPIFM/without RTPIFM group.

### Application of TPUS-IGRT to hypofractionated radiotherapy

CDFRT has a long history as a curative EBRT modality for LPCa. On the other hand, Brenner and Hall proposed that the α/β ratio of prostate cancer is 1.5 Gy [35], which is as small as the late response of surrounding organs. Accordingly, EBRT with increased fraction size is also being performed.

In addition to SBRT [13, 14], moderate hypofractionated radiotherapy (m-HpoRT) with a fraction size of 2.5 Gy/fr is also used [36]. The strategy to improve the local control rate by increasing the fraction size taking advantage of the low α/β ratio of prostate cancer is attractive, and the application of TPUS-IGRT to SBRT and m-HpoRT is well worth considering.

However, in EBRT without IMRT, as in our study, the risk of developing GI late toxicity should be considered.

According to Brand et al. [37], for G1 + rectal bleeding, one of the most objective endpoints, the α/β ratio 95% confidence interval upper bound was < 3 Gy. Using this 3 Gy value, the EQD2 (equivalent dose in 2 Gy fractions) for rectal late toxicity is calculated when the rectum is irradiated with the dose prescribed for curative prostate cancer: EQD2 = 77 Gy for 70 Gy/28 fr [36] irradiation and 35–40 Gy/5 fr [13] irradiation, EQD2 = 70–88 Gy. Considering that "GI late toxicity Grade 2 or higher was significantly higher in the 78 Gy/39 fr group (in other words, above 76 Gy/38 fr)" in our study, when TPUS-IGRT is applied to SBRT and m-HpoRT, techniques to reduce the volume of rectal irradiation using IMRT may be necessary.

## Limitations of our study

This is not a randomized study with or without TPUS-IGRT, and it is not possible to accurately assess the extent to which bPFS, GU and GI toxicity changed with or without TPUS-IGRT itself.

TPUS-IGRT is performed in conditions where IMRT is not performed due to facility limitations, and there is room for IMRT concomitant use, especially to reduce GI toxicity. Viani et al. [38], in a comparative study of IMRT and three-dimensional conformal radiotherapy (3D-CRT), found no significant difference in bPFS between IMRT and 3D-CRT, but late GI toxicity was significantly lower with IMRT.

CAS is not equipped with a mechanism to record beam-on time (BOT). Our study is a retrospective study, it is not possible to obtain data on BOT, therefore BOT cannot be included in the evaluation of intraPM.

In addition, 17 of the 123 patients (13.8%) included in the analysis had a relatively short follow-up period of 2 years or less, and thus, the long-term outcome and late toxicity have not yet been sufficiently evaluated. Further follow-up is needed to confirm long-term outcomes.

## Conclusion

Under the circumstance that approximately 1/3 of the RTPIFM fractions underwent recorrection, the above mid-term results suggest that TPUS-IGRT is well tolerated in CDFRT, as the bPFS and late toxicity rates are almost comparable to those reported by other authors for CDFRT with IGRT. In the poor IR/HR/VHR comparison between the ≤ 76 Gy group and the 78 Gy group, the incidence of Grade 2 or higher GU and GI late toxicity was higher in the 78 Gy group. Special considerations may be necessary, such as dose reduction, even when TPUS-IGRT is used, especially in patients with suspected intolerance to radiation, such as those on HD.

### Supplementary Information

Supplementary Material 1.

Supplementary Material 2.

Supplementary Material 3.

Supplementary Material 4.

Supplementary Material 5.

## Data Availability

The datasets generated and/or analyzed during the current study are not publicly available due [REASON WHY DATA ARE NOT PUBLIC] but are available from the corresponding author on reasonable request.

## Abbreviations

EBRT: External beam radiotherapy
LPCa: Localized prostate cancer
bPFS: Biological prostate-specific antigen relapse-free survival rate
IntraPM: Intrafractional prostate motion
InterPM: Interfractional prostate motion
IGRT: Image-guided radiotherapy
HR: High risk
EMT: Electromagnetic transponders
Linac: Linear accelerator
MRI: Magnetic resonance imaging
CAS: Clarity autoscan system
SBRT: Stereotactic body radiotherapy
CDFRT: Conventional dose-fractionated EBRT
YCHS: Yamagata City Hospital Saiseikan
OS: Overall survival
TPUS-IGRT: Transperineal ultrasound image-guided radiotherapy
CT: Computed tomography
HTx: Hormonal therapy
PSA: Prostate-specific antigen
NCCN: National Comprehensive Cancer Network
LR: Low risk
IR: Intermediate risk
VHR: Very high risk
HD: Hemodialysis
RA: Rheumatoid arthritis
WS: Workstation
RPV: Reference positioning volume
CTV: Clinical target volume
IC: Isocenter
PTV: Planning target volume
SI: Superior-inferior side
RL: Right-left side
RTPIFM: Real-time prostate intrafraction monitoring
SAH: Subarachnoid hemorrhage
NAHT: Neoadjuvant HTx
AHT: Adjuvant HTx
CTCAE: Common terminology criteria for adverse events
GU: Genitourinary
GI: Gastrointestinal
UA: Univariate analysis
MA: Multivariate analysis
GSG: Gleason score group
ED: Erectile dysfunction
HBO: Hyperbaric oxygenation
IMRT: Intensity-modulated radiotherapy
CRF: Chronic renal failure
m-HpoRT: Moderate hypofractionated radiotherapy
3D-CRT: Three-dimensional conformal radiotherapy
BOT: Beam-on time
