# Supplementary material for: Treatment outcome of localized prostate cancer using transperineal ultrasound image-guided radiotherapy
Source: Radiat Oncol. 2024 Aug 1;19:100. doi: 10.1186/s13014-024-02490-x (PMC11292876; doi:10.1186/s13014-024-02490-x)
Supplement: Supplementary file 1 — Supplementary Material 1. [file 13014_2024_2490_MOESM1_ESM.pdf]

Supp. A1

Clarity AutoScan System (Elekta, Stockholm, Sweden) Composition

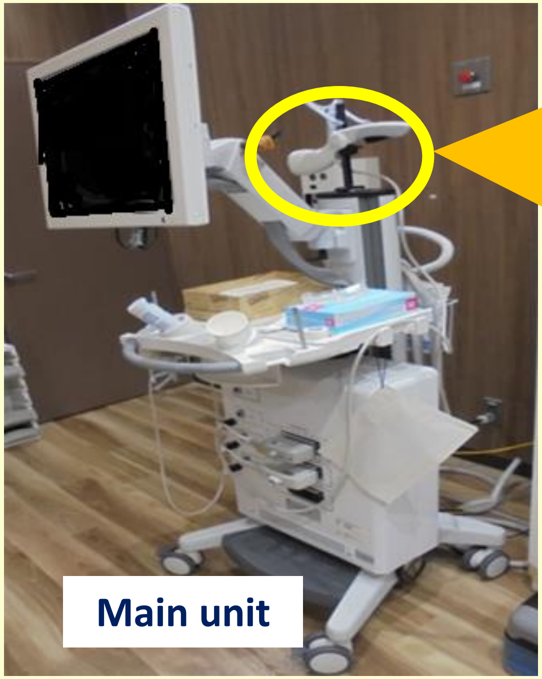

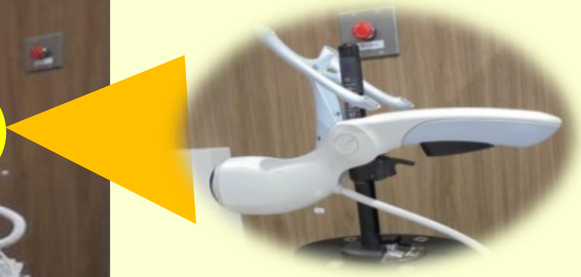

Transperineal probe

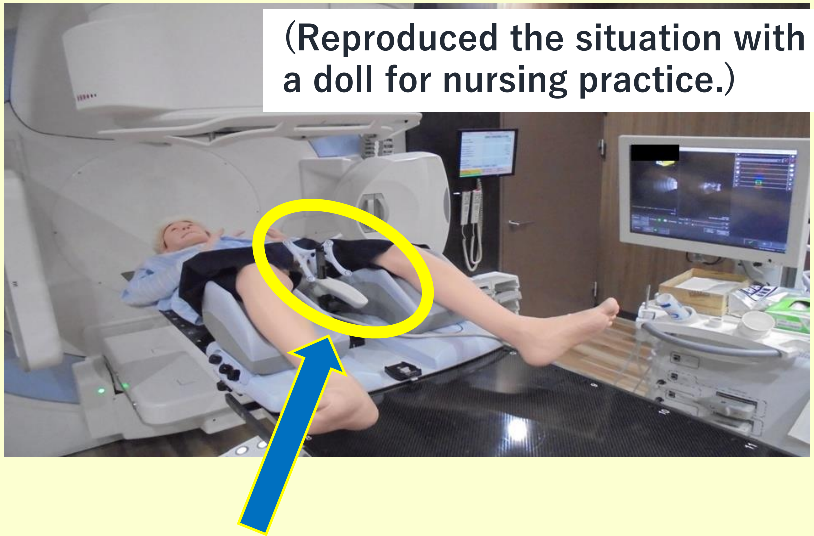

The patient is set up on the linear accelerator bed and the transperineal probe is attached. The RPV position is corrected before irradiation.

Supp. A2

Screen for RTPIFM (operational since November 21, 2017)

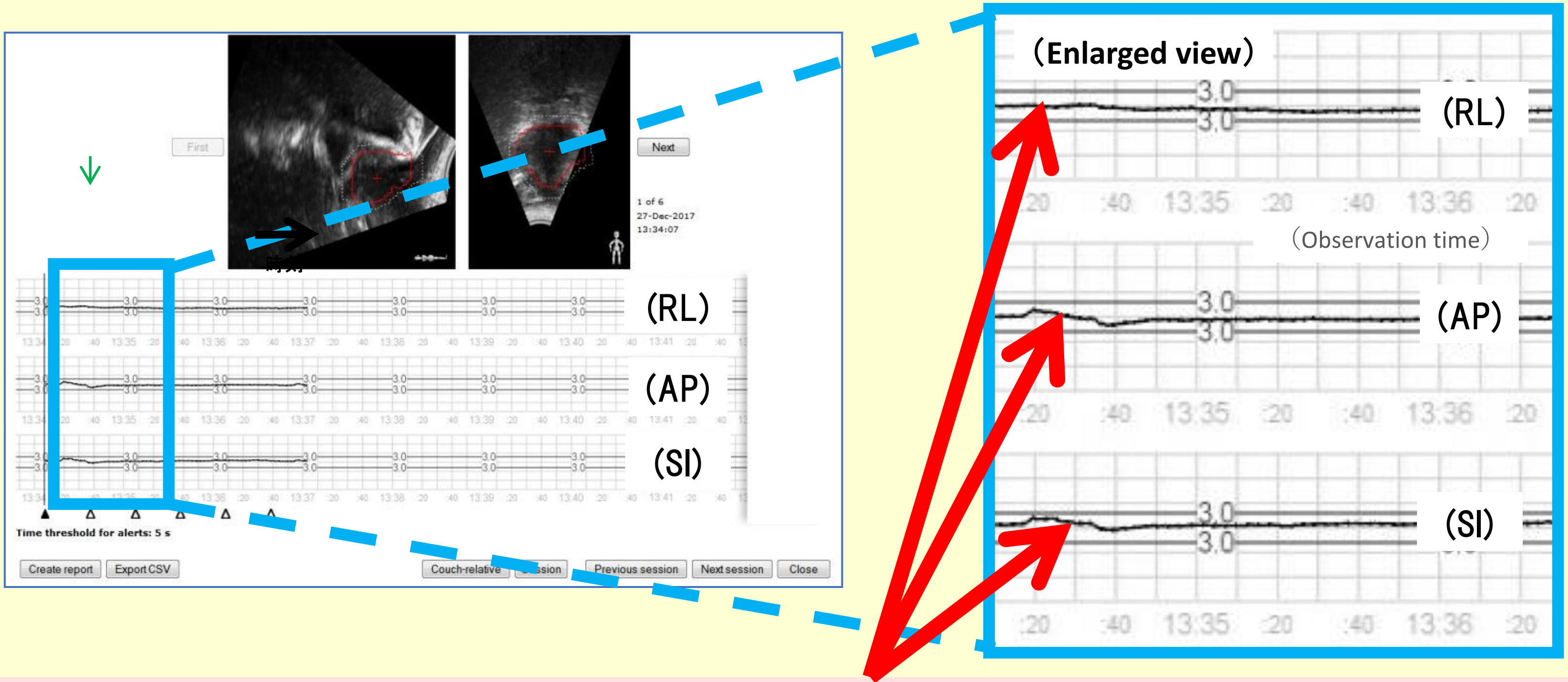

Bold lines show the relative displacement of the RPV (prostate) from the point of correction just before irradiation. If the RPV is found to be displaced more than 2-3 mm in either direction, the beam is paused and the patient is placed on standby/recorrected until reinstatement.

Abbreviation: RPV, reference positioning volume; RTPIFM, real-time prostate intrafractional monitoring; RL, right-left; AP, anterior-posterior; SI, superior-inferior.
