# Supplementary material for: Treatment outcome of localized prostate cancer using transperineal ultrasound image-guided radiotherapy
Source: Radiat Oncol. 2024 Aug 1;19:100. doi: 10.1186/s13014-024-02490-x (PMC11292876; doi:10.1186/s13014-024-02490-x)
Supplement: Supplementary file 2 — Supplementary Material 2. [file 13014_2024_2490_MOESM2_ESM.docx]

| administered dose | Dose (@isocenter) | n |
| --- | --- | --- |
| LR | 74Gy/37fr | 7 |
| good IR | 76Gy/38fr | 24 |
|  | 74Gy/37fr | 1* |
| poor IR | 76Gy/38fr& | 27 |
|  | 78Gy/39fr$ | 14 |
|  | 68.4Gy/38fr | 1☆ |
| poor IR | 76Gy/38fr& | 27 |
|  | 78Gy/39fr$ | 14 |
|  | 68.4Gy/38fr | 1☆ |
| HR/VHR | 76Gy/38fr& | 19 |
|  | 78Gy/39fr$ | 30 |
|  | 32Gy/16fr | 1♠ |

Supp. B Administered Dose

& -Jun. 2018

$ Jul 2018 –

*Initially, 74 Gy was administered with a diagnosis of LR. Changed to favorable IR on reassessment.

☆Dose reduced due to rheumatoid arthritis.

♠Discontinued at 32 Gy/16 fr due to the onset of subarachnoid hemorrhage.

Abbreviations as in Table1.
