# Supplementary material for: Treatment outcome of localized prostate cancer using transperineal ultrasound image-guided radiotherapy
Source: Radiat Oncol. 2024 Aug 1;19:100. doi: 10.1186/s13014-024-02490-x (PMC11292876; doi:10.1186/s13014-024-02490-x)
Supplement: Supplementary file 3 — Supplementary Material 3. [file 13014_2024_2490_MOESM3_ESM.docx]

Supp. C HTx details

|  |  | n | % |
| --- | --- | --- | --- |
| NAHT | LR | 1 | 14.3 |
|  | favorable IR | 15 | 60.0 |
|  | poor IR | 33 | 78.6 |
|  | HR | 35 | 89.7 |
|  | VHR | 11 | 100.0 |
|  | Total | 95 | - |
| PSA just before EBRT (ng/ml) | <1.0 | 84 | 67.7 |
|  | ≧1.0 | 39§ | 31.5 |
|  | Unknown | 1 | 0.8 |
| HTCRT | Yes | 93 | 75.0 |
| AHT Median 13M(1-65M) | None | 38 | 30.6 |
|  | 1-6M | 27 | 21.8 |
|  | >6M | 58 | 46.8 |
|  | unknown | 1 | 0.8 |
|  | Total | 124 | 100 |

Abbraviations: HTx, hormonal therapy; NAHT, neoadjuvant HTx ; HTCRT, HTx concurrent with radiotherapy ; AHT, adjuvant HTx; M, months.

Others are the same as in Table 1.

§Includes 29 patients who did not undergo NAHT.
