# Supplementary material for: Treatment outcome of localized prostate cancer using transperineal ultrasound image-guided radiotherapy
Source: Radiat Oncol. 2024 Aug 1;19:100. doi: 10.1186/s13014-024-02490-x (PMC11292876; doi:10.1186/s13014-024-02490-x)
Supplement: Supplementary file 4 — Supplementary Material 4. [file 13014_2024_2490_MOESM4_ESM.docx]

Supp. D1

bPFS

76Gy 96.4%(4y)

78Gy 85.7%(4y)

p=0.5169 (Logrank test)

Abbreviations same as Figure 1.
