# Supplementary material for: Treatment outcome of localized prostate cancer using transperineal ultrasound image-guided radiotherapy
Source: Radiat Oncol. 2024 Aug 1;19:100. doi: 10.1186/s13014-024-02490-x (PMC11292876; doi:10.1186/s13014-024-02490-x)
Supplement: Supplementary file 5 — Supplementary Material 5. [file 13014_2024_2490_MOESM5_ESM.docx]

Supp. D2

bPFS

76Gy 89.5%(4y)

78Gy 95.0%(4y)

p=0.3769 (Logrank test)

Abbraviations same as Figure 1.
